# Extracts of *Dracocephalum tanguticum* Maxim Ameliorate Acute Alcoholic Liver Disease *via* Regulating Transcription Factors in Mice

**DOI:** 10.3389/fphar.2022.830532

**Published:** 2022-03-16

**Authors:** Min Guo, Liwei Gu, Heping Hui, Xiaodong Li, Ling Jin

**Affiliations:** ^1^ College of Pharmacy, Gansu University of Chinese Medicine, Lanzhou, China; ^2^ Institute of Chinese Materia Medica, Gansu Academy of Traditional Chinese Medicine, Lanzhou, China; ^3^ Laboratory of Chinese Medicine, Gansu Provincial Hospital of Traditional Chinese Medicine, Lanzhou, China; ^4^ Institute of Chinese Materia Medica, China Academy of Chinese Medical Sciences, Beijing, China; ^5^ Gansu Agriculture Technology College, Lanzhou, China

**Keywords:** *Dracocephalum tanguticum* Maxim, acute alcoholic liver disease, transcription factors, Tibetan medicine, extract

## Abstract

Alcoholic liver disease (ALD) caused by excessive drinking is a health and economic concern worldwide. Given the high morbidity, mortality, and the progressive nature of ALD, finding effective interventions is essential. Previous studies have confirmed that edible food plants and their bioactive compounds exert a protective effect against ALD. *Dracocephalum tanguticum* Maxim (*DTM*) is one of the important traditional Tibetan medicines in China with the effect of clearing away liver heat, used for the treatment of hepatitis. In this study, the *DTM* chloroform extract (*DtM-C*), ethyl acetate extract (*DtM-E*), and n-butanol extract (*DtM-B*) were obtained by ethanol extraction combined with fractional extraction. Acute ALD was induced in mice given intragastric ethanol. Serum and liver biochemical markers were detected by ELISA. Liver histological observation, Oil Red O, and Masson’s trichrome staining were performed. Liver injury cells were induced by ethanol. The cell vitality was detected by using MTT colorimetry. The expressions of Nrf2, NF-κB, STAT3, AP-1, CREB, HIF-1α, HO-1, NQO-1, GSTA1, IKB2, and Keap1 were detected by real-time polymerase chain reaction (PCR) to elucidate the mechanism of hepatoprotective effect, and the results were verified by using Western blot. The results of serum liver function indicators (ALT, AST, and ADH), serum hepatic lipid indicators (TC, TG, HDL-C, and LDL-C), and lipid peroxidation indicators (ADH, MDA, SOD, CAT, and GSH-Px) in liver tissue and liver histological observation showed that *DtM-E* could improve liver function, alleviate fatty degeneration, edema, cell necrosis, and liver fibrosis caused by alcohol. *DtM-E* also increased the vitality of EtOH-induced liver injury cells, upregulated the mRNA expression of Nrf2, HO-1, NQO-1, and GSTA1, while downregulated the expression of Keap-1, p65, and NF-κB. Western blot results were consistent with PCR. The results suggest that *DtM-E* has a protective effect against ALD *in vitro* and *in vivo*, and its mechanism of action may be related to the activation of Nrf2/Keap-1 and inhibition of the P65/NF-κB signaling pathways.

## Introduction

Alcoholic liver disease (ALD) represents a spectrum of injury, ranging from steatosis (fatty liver) to alcoholic hepatitis (AH, a combination of steatosis and inflammation) to cirrhosis and hepatocellular carcinoma (Stickel et al., 2017; Singal et al., 2018). ALD is the leading cause of alcohol-related deaths worldwide, producing huge health and economic burden (Alcohol and Collaborators, 2018). Alcohol consumption is a major risk factor (Osna et al., 2017; Asrani et al., 2019). According to the World Health Organization, the highest levels of per capita alcohol consumption are observed in countries of the European Region (Dunn and Shah, 2016; Seitz et al., 2018; Sharma and Arora, 2020). Recently, alcohol consumption is also alarmingly increasing in developing countries (Kim et al., 2016). With explosive economic growth and increasing social openness in China (Zhang et al., 2018), the alcohol consumption has significantly boosted over the last 30 years. At 2013, China was globally ranked as the second heaviest drinking country, and with it comes the incidence of ALD (Wang et al., 2019).

Other than cessation of alcohol use, medical therapy of ALD is limited (Singh et al., 2017). Abstinence is still the main therapy for all stages of ALD. In the earliest simple steatosis stage, ALD can be reversed by alcohol abstinence. For patients with hepatitis, corticosteroids are the main therapy (Marroni et al., 2018). When the disease progresses to decompensated cirrhosis, liver transplantation is the only definitive therapy. However, scarcity of organ donation, high operational costs, and immune rejection make patients with ALD helpless in the end stage. Therefore, it is urgent to develop safer and more effective therapeutic methods.

The accumulating evidence has shown that treatment with natural plant extractions for ALD has gained attention (Ding et al., 2012). A variety of natural medicines from herbal plants to traditional Chinese medicine formula have shown antioxidation and anti-inflammatory effects and the ability to regulate fat metabolism. Their curative effect has progressively been recognized.

*Dracocephalum tanguticum* Maxim (DTM) is one of the important traditional Tibetan medicines, also named “zhiyangge,” which has been recorded in the Tibetan medicine standard (volume 1). *DTM* is mainly distributed in Northeast Asia and the high-altitude mountain areas in western China. Related studies reported that it contains triterpenoids, steroids, flavonoids, alkaloids, lignans, and coumarins. Previous pharmacological studies have shown them with antioxidant, antihypoxic, antitumor, immunomdulatory, and antimicrobial biological activities. It is one of the most common Tibetan medicines used for the treatment of hepatitis, gastritis, dizziness, arthritis, and ulcer (Ye et al., 1994; Wang et al., 2010; Xu et al., 2011). Tibet is located in the high and cold region. To keep warm and the ethnic culture, people often drink a lot of alcohol. Years of observation found that local patients with ALD are rare. However, no study has been reported on *DTM* for the treatment of ALD.

This study aims to observe the amelioration of DTM chloroform extract (DtM-C), ethyl acetate extract (DtM-E), and n-butanol extract (DtM-B) against ALD *in vitro* and *in vivo*, screen the active site, explore the engaged mechanism based on the transcription factors, and provide a new therapeutic method for ALD.

## Materials and methods

### Materials

Assay kits for catalase (CAT, A007-1-1), malondialdehyde (MDA, A003-1-2), superoxide dismutase (SOD, A001-3-2), alcohol dehydrogenase (ADH, A083-1-1), glutathione peroxidase (GSH-Px, A005-1-2), alanine transaminase (ALT, C009-2-1, aspartate transaminase (AST, C010-2-1), total cholesterol (TC, A111-1-1), triglyceride (TG, A110-1-1), low-density lipoprotein cholesterol (LDL-C, A113-1-1), high-density lipoprotein cholesterol (HDL-C, A112-1-1) and hematoxylin–eosin staining kits were purchased from Nanjing Jiancheng Bioengineering Institute (Nanjing, Jiangsu, China). BCA Protein Quantitative Kit was provided by Conway Century Biotechnology Co., Ltd., DMEM culture medium dry powder was from Gibco Company, USA; fetal bovine serum was from Gibco Company, USA; methyl thiazolyl tetrazolium salt (MTT) was from Amresco Company, USA; dimethyl sulfoxide (DMSO) was from Sigma, USA; Total RNA Extraction Kit (centrifugal column type), First-Chain cDNA Synthesis Kit, and SuperReal Fluorescence Quantitative Suspension Kit (SYBR Green) were purchased from Tiangen Biochemical Technology (Beijing) Co., Ltd. RIPA lysate was from Solarbio, China; 0.45 μm of VDF membrane was from Millipore, USA; acrylamide, bis-acrylamide, APS, TEMED, and Bromphenol Blue were from Amresco, USA; glycine, sodium dodecyl sulfate (SDS), and Trizma base were from Sigma, USA; and β-actin was from ImmunoWay, YM3028, USA. The primary antibodies against Nrf2(ab62352), HO-1 (ab52947), NQO-1 (ab80588), GSTA1(ab180650), Keap1 (ab66620), IKB2(ab109509), and NF-κB p65 (ab76302) were purchased from Abcam (UK).

The primers were synthesized by Invitrogen (Shanghai) Trading Co., Ltd. All other reagents were of analytic grade. Silibinin capsules were obtained from Tianshili Pharmaceutical Group Co., Ltd.

### Preparation of chloroform extract, n-butanol extract, and ethyl acetate extract

Dried *DTM* was purchased from Qizheng Group Gannan Foge Tibetan Medicine Co., Ltd., and identified by *Ling Jin*, a Professor in the Department of Chinese Pharmacognosy, Gansu University of Chinese Medicine. The medicinal materials were cut into 2-cm-long segments. Each gram of medicinal materials was soaked in 75% ethanol for 20 ml for 15 days. The extracts were filtered, and the liquid was extracted with chloroform, ethyl acetate, and *n*-butanol, respectively. After making the extracts concentrated using a rotary evaporator, *DtM-C*, *DtM-B*, and *DtM-E* were obtained, and the extraction rates were 1.35%, 7.92%, and 1.44%, respectively.

### *In vivo* hepatoprotective effects of extracts from *Dracocephalum tanguticum* Maxim on Et-OH-induced acute alcoholic liver disease mice

#### Animals and treatments

Sixty male ICR mice (20–22 g, 6- to 8-week old) were provided by the Laboratory Animal Center of Lanzhou University [Production license No. SCXK-(Gansu)2018-0002] and housed in a specific environment (22°C ± 2°C, 60% ± 5% relative humidity) under 12-h light–dark cycles. The animal protocol was approved by the Animal Experimentation Ethics Committee of the Gansu University of Chinese Medicine (approval No. 2019-084).

The method was modified binge ethanol administration (Dastidar et al., 2018). The mice were randomly divided into six groups (*n* = 10): normal control group (NC), model group, positive drug group, *DtM-C* group, *DtM-B* group, and *DtM-E* group. Except the NC group, the mice in the other groups were fasted for 16 h and treated with 40% v/v alcohol at a dose of 17 ml/kg/12 h three times to establish the acute ALD mice model. Based on the body surface calculated according to the Meeh–Rubner formula (Chen., 2011), extraction rate, and clinical dosage (Ministry of Health of the People’s Republic of China., 1995), the positive drug group, *DtM-C* group, *DtM-B* group, and *DtM-E* group were treated with silibinin capsules (63.7 mg/kg/day), *DtM-C* (48.6 mg/kg/day), *DtM-B* (285.1 mg/kg/day), and *DtM-E* (51.9 mg/kg/day), respectively, before alcohol administration for 7 days. The control and model groups were administered with an equal volume of distilled water instead of drugs.

After the last gavage, all mice were fasted for 16 h and anesthetized. The blood samples were collected into heparin-containing tubes. The serum was separated by centrifugation at 3,500 rpm for 15 min and frozen at −80°C for biochemical assay. The liver tissue was collected for further experiments.

#### Biochemical analysis

Hepatic lobule homogenate was isolated, and the supernatant was collected. The activities of ADH, SOD, MDA, CAT, GSH-Px in it and the levels of AST, ALT, ADH, TC, TG, HDL-C, LDL-C in the serum were determined following the manufacturer’s instructions for the respective kits.

#### Histopathological evaluation

Liver tissues were fixed in 4% paraformaldehyde, then embedded in paraffin solution, cut into 5-μm-thick slices, and stained with H&E for histopathological analysis of liver injury and Masson’s trichrome to evaluate hepatic collagen.

Liver tissues were frozen in liquid nitrogen, cut into 5-μm-thick sections, and stained with oil red O staining for observation of lipid accumulation.

The stained samples were observed under a light microscope (Olympus CX31, Japan).

### *In vitro* hepatoprotective effects of extracts from *Dracocephalum tanguticum* Maxim on cells

#### EtOH-induced cytotoxicity

Human cells [obtained from the Institute of Basic Medical Sciences of Chinese Academy of Medical Sciences (Beijing, China)] were kept in RPMI-1640 medium containing 10% (v/v) FBS, 100 U/ml of penicillin, and 100 mg/ml of streptomycin. Cells were seeded into 96-well plates (8 × 10^3^ cells/well) and nurtured in a 5% CO_2_ incubator at 37°C for 24 h. Then cells were incubated with ethanol (0, 25, 50, 100, 200, and 400 μM) for 24 h. After the treatments, cells were further incubated with 0.5% MTT for 4 h, then dissolved in 200 μl of DMSO, and the optical density was measured at 490 nm.

#### Effect of n-butanol extract, chloroform extract, and ethyl acetate extract on normal and EtOH-induced cells

Human hepatic LO2 cells are widely used in hepatocyte model studies *in vitro* (Shi et al., 2021). The cells were seeded into 96-well plates (8 × 10^3^ cells/well) and divided into four groups: normal control group (NC), *DtM-B* group, *DtM-C* group, and *DtM-E* group. Except the NC group, the cells in the other groups were treated with 20 μl of *DtM-B*, *DtM-C*, and *DtM-E*, respectively, at the concentrations of 5, 10, 20, 40, 80, and 160 μg/ml.

Based on the previous results, 200 μM ethanol was selected to build the alcoholic liver injury cell model. The EtOH-induced cells were divided into four groups: the model group, *DtM-B* group, *DtM-C* group, and *DtM-E* group. Except the model group, the cells in the other groups were treated with 20 μl of *DtM-B*, *DtM-C*, and *DtM-E*, respectively, at the concentrations of 5, 10, 20, 40, 80, and 160 μg/ml.

Of the extracts, 180 μl was added, and all cells were kept in a 5% CO_2_ incubator for 24 h. After the treatments, cells were further incubated with 0.5% MTT for 4 h, then dissolved in 200 μl of DMSO, and the optical density was measured at 490 nm to calculate the survival rate of each group.

#### Real-time polymerase chain reaction analysis of nuclear transcription factors

Based on the previous results, 200 μM ethanol and *DtM-E* were selected for PCR analysis of Nrf2, NF-κB, STAT3, AP-1, CREB, and HIF-1α. Cells were seeded into six-well plates (16 × 10^4^ cells/well) and divided into three groups: ① normal control group (NC); ② model group, treated with 200 μM ethanol; and ③ EtOH + *DtM-E* (40 μg/ml) group, treated with 200 μM ethanol and 40 μg/ml of *DtM-E*. All cells were nurtured in a 5% CO_2_ incubator for 24 h, and the total RNA was extracted by Trizol regents. RNA content was determined by a trace photometer (NanoDrop ND-1000, USA). cDNA was synthesized by the kit. Real-time PCR was performed, and relative expression of target genes was normalized and analyzed by the 2^−ΔΔCt^ method as ratio compared with the NC group. The primer sequences used in this study are listed in Table 1.

**TABLE 1:** List of primers for real-time polymerase chain reaction (PCR).

Target	Full gene name	Primer	Sequence (5′→3′)
GAPDH	Glyceraldehyde-3-phosphate dehydrogenase	FP	GAG​TCA​ACG​GAT​TTG​GTC​GT
		RP	GAC​AAG​CTT​CCC​GTT​CTC​AG
Nrf2	Nuclear factor-erythroid 2-related factor 2	FP	GCA​CAT​CCA​GTC​AGA​AAC​CA
		RP	GCC​GAA​GAA​ACC​TCA​TTG​TC
NF-kB p65	Nuclear factor kappa-B p65	FP	GGA​GCA​CAG​ATA​CCA​CCA​AGA
		RP	CGG​CAG​TCC​TTT​CCT​ACA​AG
STAT3	Signal transducer and activator of transcription 3	FP	TGG​AGG​AGA​GAA​TCG​TGG​AG
		RP	TTT​GAC​CAG​CAA​CCT​GAC​TTT
AP-1	Activator protein 1	FP	TGA​CTG​CAA​AGA​TGG​AAA​CG
		RP	CAG​GTT​CAG​GGT​CAT​GCT​C
CREB	cAMP-response element-binding protein	FP	AAC​CAG​CAG​AGT​GGA​GAT​GC
		RP	AGT​TAC​GGT​GGG​AGC​AGA​TG
HIF-1α	Hypoxia-inducible factor 1-alpha	FP	GCA​GCA​ACG​ACA​CAG​AAA​CT
		RP	AGC​GGT​GGG​TAA​TGG​AGA​C
HO-1	Heme oxygenase 1	FP	TCC​GAT​GGG​TCC​TTA​CAC​TC
		RP	TAA​GGA​AGC​CAG​CCA​AGA​GA
NQO-1	NAD(P)H quinone dehydrogenase 1	FP	GGG​TCC​AGT​CCC​ATG​ACC​TT
		RP	TCC​CCA​TGT​TTG​TCA​GTG​AA
GSTA1	Glutathione S-transferase alpha 1	FP	GCC​ATC​CTG​TGC​TAC​ATT​GC
		RP	CAG​CTG​ATT​GGA​GAC​GTC​CAT
Keap1	Kelch-like ECH-associated protein 1	FP	GCT​GTC​CTC​AAT​CGT​CTC​CT
		RP	ATT​CGC​CAC​TCG​TTC​CTC​T

#### Effects of ethyl acetate extract on the Nrf2/Keap-1 signaling pathway

PCR analysis was used to detect the expression of Keap-1, Nrf2, HO-1, NQO-1, and GSTA1mRNA. The method was the same as the PCR analysis of nuclear transcription factors.

#### Effects of ethyl acetate extract on the IKB2/NF-κB signaling pathway

PCR analysis was used to detect the expression of IKB2, NF-κB, and NF-κB P65mRNA. The method was the same as the PCR analysis of nuclear transcription factors.

#### Western blotting

Total cytoplasmic and nuclear proteins were obtained from the cells using protein extraction kits. The protein content was determined by the BCA protein assay kit. An equivalent amount of protein (20 μg) was then separated by 12% SDS-PAGE and transferred into polyvinylidene difluoride membranes (PVDFs). The membranes were probed with primary antibodies against β-actin (1:2,000 dilutions) and secondary antibodies (1:10,000 dilutions) and were further visualized by using enhanced chemiluminescent reagents. The gray densities of the protein bands (Nrf2, HO-1, NQO-1, GSTA1, Keap1, IKB2, NF-κB p65) were normalized by using β-actin as internal controls, and analyzed by the Alphaview SA software (Fluor Chem FC3, USA).

### Statistical analysis

Graphs were generated using GraphPad Prism 6.0. Statistical analysis was performed using the SPSS 22.0 software, and the data were expressed as mean ± standard deviation. One-way ANOVA with Tukey’s *post-hoc* test was employed to assess the multiple comparisons when there was no variance in homogeneity; otherwise, Dunnett T3 was performed for analysis among groups. A value of *p *< 0.05 is considered statistically significant.

## Results

### *In vivo* hepatoprotective effects of extracts from *Dracocephalum tanguticum* Maxim on Et-OH-induced acute alcoholic liver disease mice

#### Results of biochemical indicator detection in serum

**FIGURE 1:**
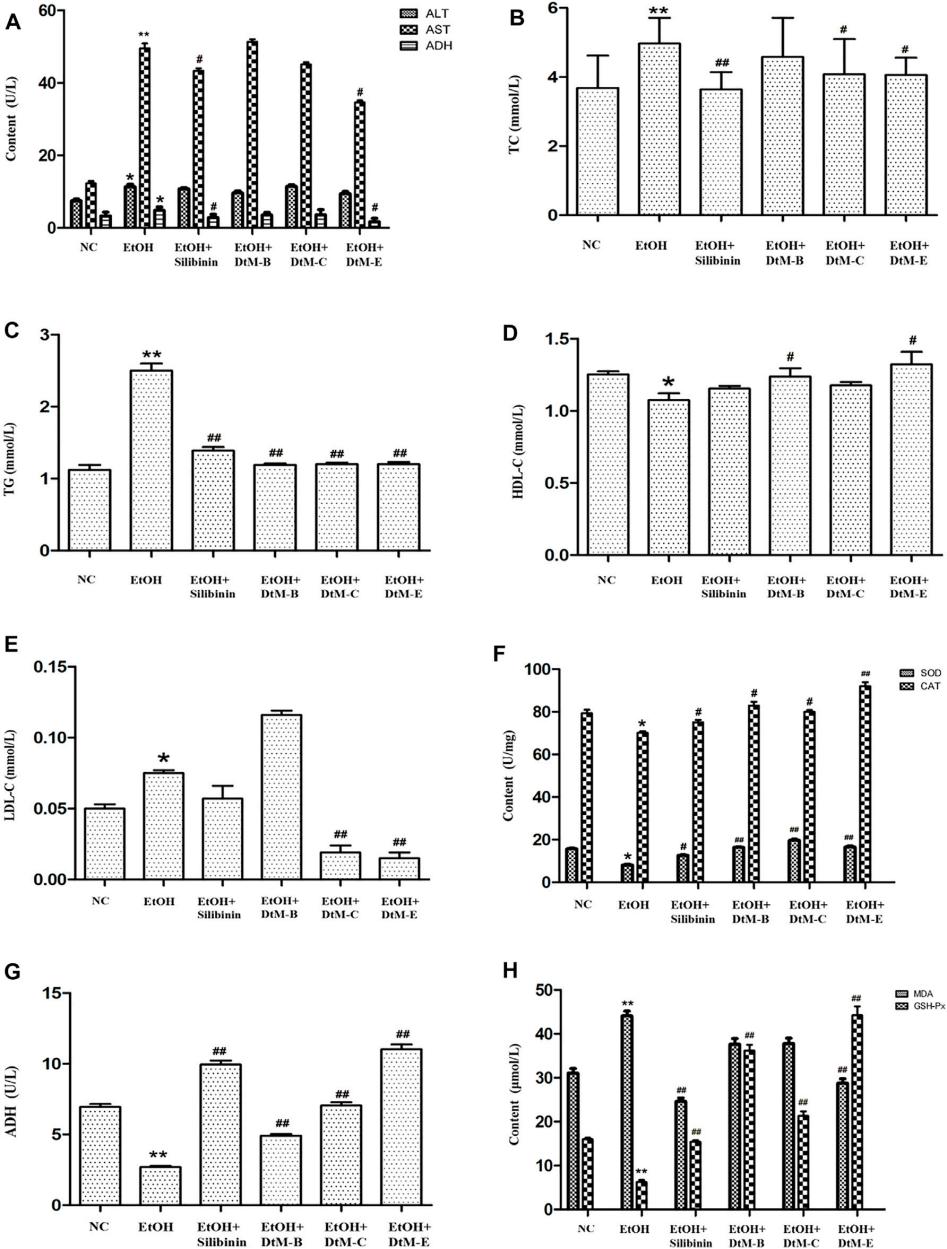
Results of the detection of biochemical indicators. **(A)** Detection of serum liver function indicators: alanine transaminase (ALT), aspartate transaminase (AST), and alcohol dehydrogenase (ADH). **(B–E)** Detection of serum hepatic lipids: total cholesterol (TC), triglyceride (TG), high-density lipoprotein cholesterol (HDL-C), and low-density lipoprotein cholesterol (LDL-C). **(F–H)** Detection of lipid peroxidation: alcohol dehydrogenase (ADH), malondialdehyde (MDA), superoxide dismutase (SOD), catalase (CAT), and glutathione peroxidase (GSH-Px) in liver tissue. All data are presented as mean ± SD (*n* = 10). *p < 0.05 and **p < 0.01 (*vs*. control group); ^#^ *p *< 0.05 and ^##^ *p *< 0.01 (*vs*. model group).

The results of serum liver function indicator in mice (Figures 1A–E) showed that compared with the NC group, alcohol could increase the contents of ALT, AST, ADH, TC, TG, and LDL-C, and decrease the content of HDL-C. The intervention of silibinin and *DtM-E* could reduce the contents of AST and ADH. Silibinin and all extracts of *DtM* could reduce the content of TG. Silibinin, *DtM-C*, and *DtM-E* could reduce the contents of TC and LDL-C. *DtM-B* and *DtM-E* could increase the content of HDL-C.

#### Results of liver lipid peroxidation in liver tissue

The results of lipid peroxidation in liver tissue (Figures 1F–H) showed that compared with the NC group, alcohol could decrease the activities of SOD, CAT, ADH, and GSH-Px, and increase the activity of MDA. The intervention of silibinin could increase the activities of SOD, CAT, ADH, and GSH-Px, and reduce the activity of MDA. *DtM-B* could increase the activities of SOD, CAT, ADH, and GSH-Px. *DtM-C* could increase the activities of SOD, CAT, ADH, and GSH-Px. *DtM-E* could increase the activities of SOD, CAT, ADH, and GSH-Px; otherwise, it could reduce the activity of MDA.

#### Results of pathological observation

**FIGURE 2:**
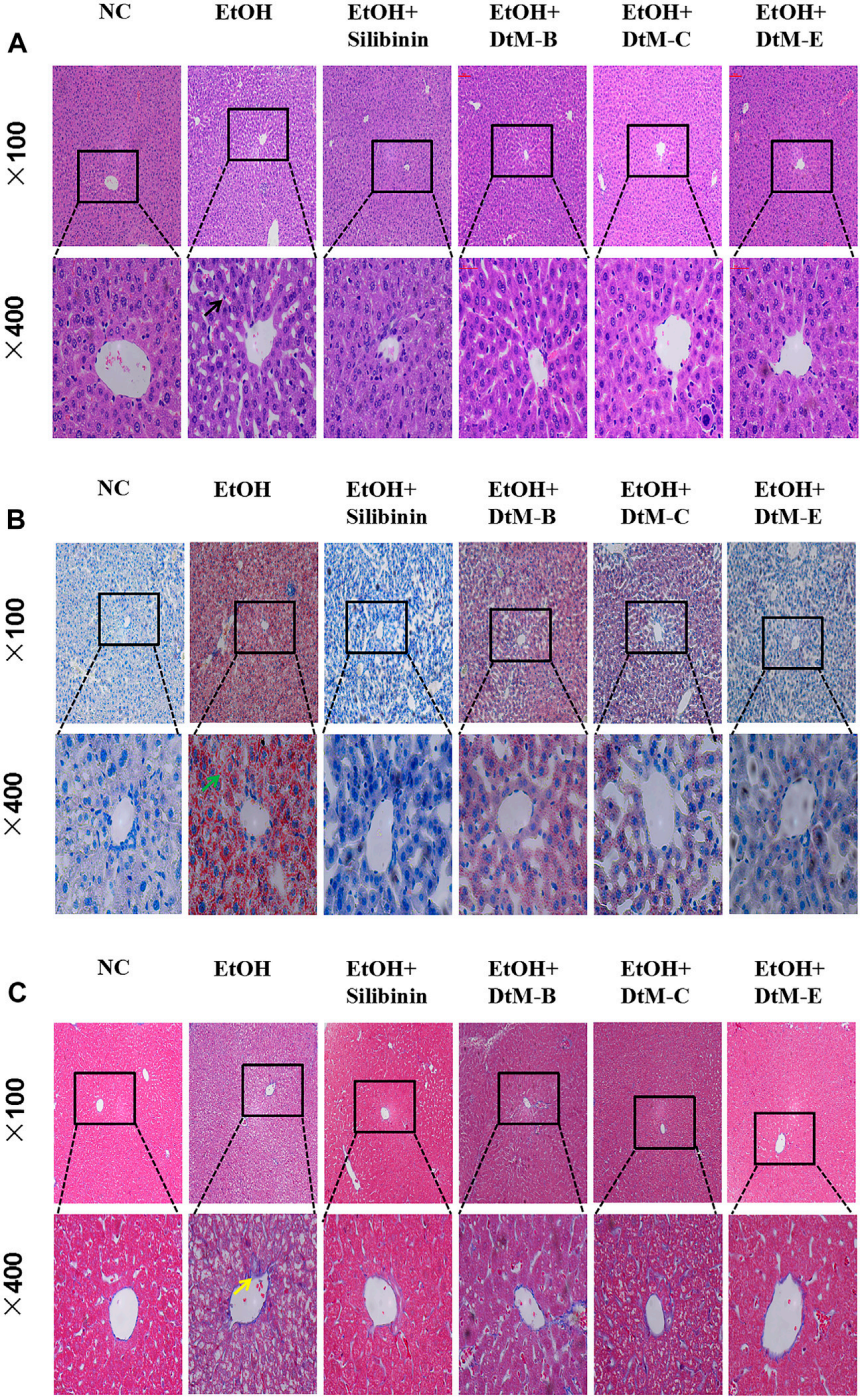
Histological photomicrographs of liver sections stained with hematoxylin and eosin (H&E) **(A)**, oil red O **(B)**, and Masson’s trichrome **(C)**; cell necrosis (black arrow), fatty degeneration (green arrow), fibrosis (yellow arrow).

The results (Figure 2) showed that in the NC group, the hepatic lobules were well organized and radially arranged. The cells were well demarcated and regular in shape. There were no hepatic cell necrosis, lymphocyte infiltration, obvious fatty degeneration, and fibrosis. Compared with the NC group, the model group exhibited enlargement of hepatocytes, steatosis, and fibrosis, which are represented by vacuolation, microvesicular fat droplets, and collagenous fiber and lymphocyte infiltration in hepatocytes from the central hepatic vein and hepatic lobule margin. In the *DtM-B* group, the structure of hepatic lobules was basically preserved, and focal hepatocyte ballooning degeneration was observed. Hepatic cell necrosis and focal lymphocyte infiltration were occasionally observed in hepatic lobules. In the *DtM-C* group, the hepatic lobule structure was clear, hepatocytes showed diffuse mild-to-moderate granular degeneration, the focal hepatocyte was steatosis, and there was no obvious balloon degeneration. Occasional punctate necrosis of hepatocytes and lymphocyte infiltration were observed in the lobules. The pathological changes in the liver in the groups of *silibinin* and *DtM-E* were similar to those in the NC group.

### *In vitro* hepatoprotective effects of extracts from *Dracocephalum tanguticum* Maxim on cells

#### EtOH-induced cytotoxicity

**FIGURE 3:**
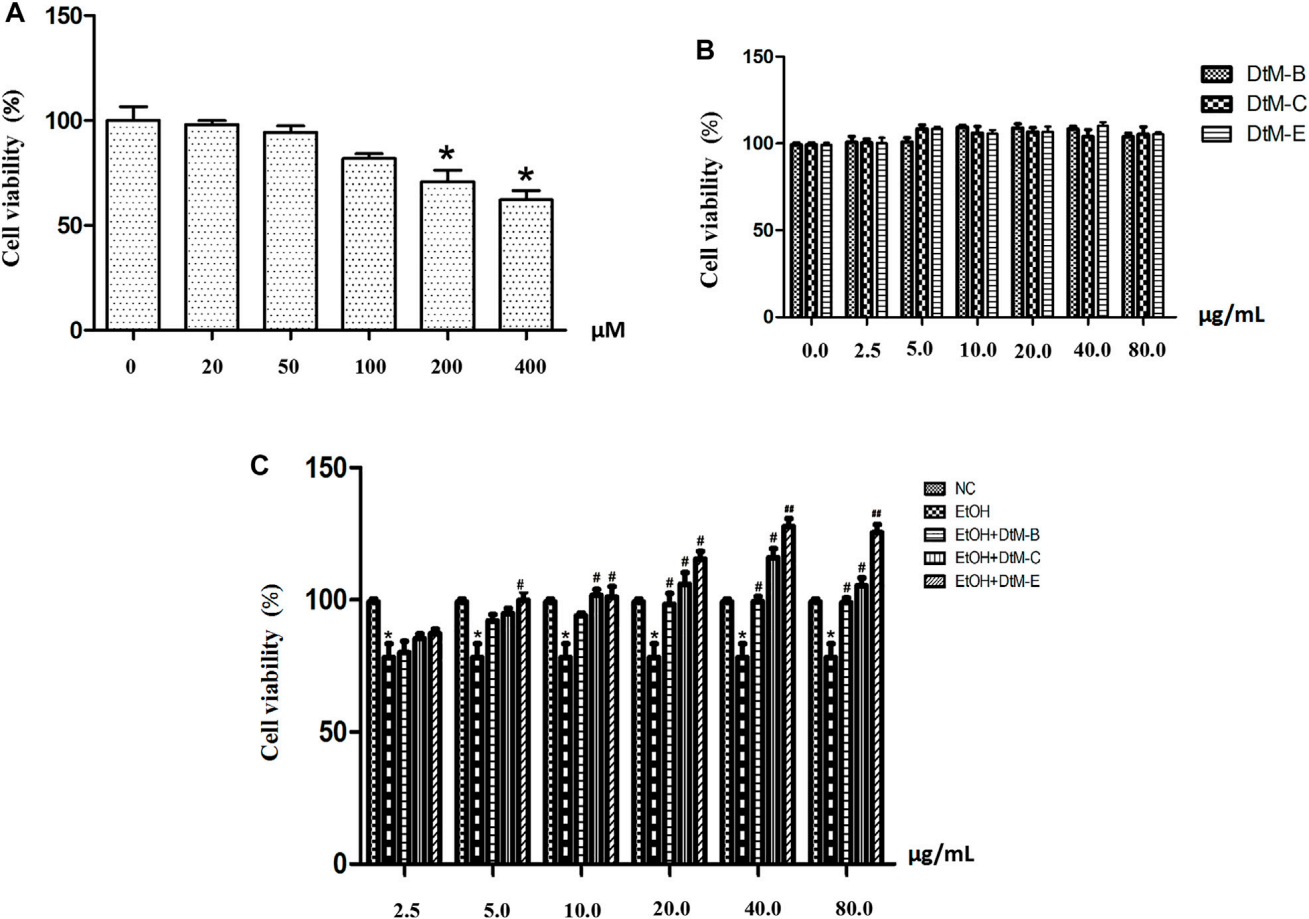
EtOH-induced cytotoxicity at different concentrations **(A)** and the effect of n-butanol extract (DtM-B), chloroform extract (DtM-C), and ethyl acetate extract (DtM-E) on normal **(B)** and Et-OH induced **(C)** cells. All data are presented as mean ± SD (*n* = 6). *p < 0.05 (*vs*. control group); *p *< 0.05 and ^##^ *p *< 0.01 (*vs*. model group).

An EtOH-induced alcoholic liver injury cell model was established. As shown in Figure 3A, at concentrations of 25 μM (98.10 ± 1.79) and 50 μM (94.28 ± 3.21), the cell viability remained almost unchanged compared with that in the control group. The cell viability was reduced at concentrations of 100 μM (81.98 ± 2.22), 200 μM (70.77 ± 5.53), and 400 μM (62.20 ± 4.38).

#### Effects of n-butanol extract, chloroform extract, and ethyl acetate extract on normal and Et-OH-induced cells

The results (Figure 3B) showed that *DtM-B*, *DtM-C*, and *DtM-E* had no significant effect on the viability of normal cells. The results (Figure 3C) showed that compared with the NC group, alcohol could decrease the viability of normal cells. Compared with the model group, at a concentration of 2.5 μg/ml, the cell viability remained almost unchanged. At a concentration of 5.0 μg/ml, *DtM-E* could increase the cell viability. At a concentration of 10.0 μg/ml, *DtM-C* and *DtM-E* could increase cell viability. At a concentration of 20.0 μg/ml, *DtM-B*, *DtM-C*, and *DtM-E* could increase cell viability. At the concentrations of 40.0 and 80.0 μg/ml, *DtM-B*, *DtM-C*, and *DtM-E* could increase cell viability.

#### Effects of ethyl acetate extract on nuclear transcription factors of Nrf2, NF-κB, STAT3, AP-1, CREB, and HIF-1α

**FIGURE 4:**
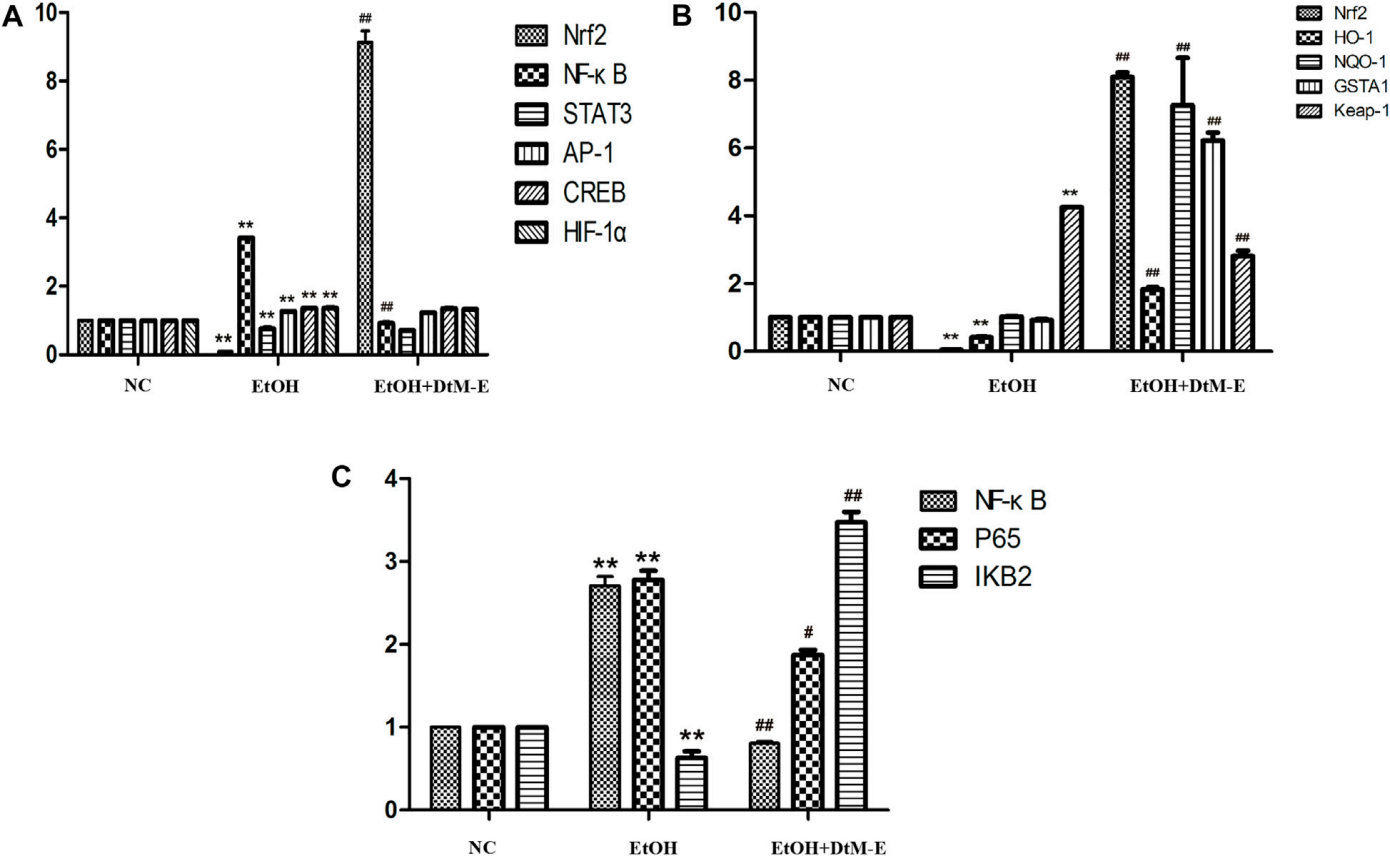
Effects of DtM-E on nuclear transcription factors of Nrf2, NF-κB, STAT3, AP-1, CREB, and HIF-1α **(A)**, the signaling pathways of Nrf2/HO-1**(B)**, and P65/NF-κB **(C)**. All data are presented as mean ± SD (*n* = 6). **p < 0.01 (*vs*. control group); *p *< 0.01 (*vs*. model group).

The results (Figure 4A) showed that compared with the NC group, alcohol downregulated the expression of Nrf2 and STAT3, while it upregulated the expression of NF-κB, AP-1, CREB, and HIF-1α. Compared with the model group, *DtM-E* upregulated the expression of Nrf2 and downregulated the expression of NF-κB.

#### Effects of ethyl acetate extract on the Nrf2/Keap-1 signaling pathway

The results (Figure 4B) showed that compared with the NC group, alcohol downregulated the expression of Nrf2 and HO-1, while it upregulated the expression of Keap-1. Compared with the model group, *DtM-E* upregulated the expression of Nrf2, HO-1, NQO-1, and GSTA1 and downregulated the expression of Keap-1.

#### Effects of ethyl acetate extract on the IKB2/NF-κB signaling pathway

The results (Figure 4C) showed that compared with the NC group, alcohol upregulated the expression of NF-κB and P65, while it downregulated the expression of IKB2. Compared with the model group, *DtM-E* downregulated the expression of NF-κB and NF-κB P65, while it upregulated the expression of IKB2.

#### Results of Western blot

**FIGURE 5:**
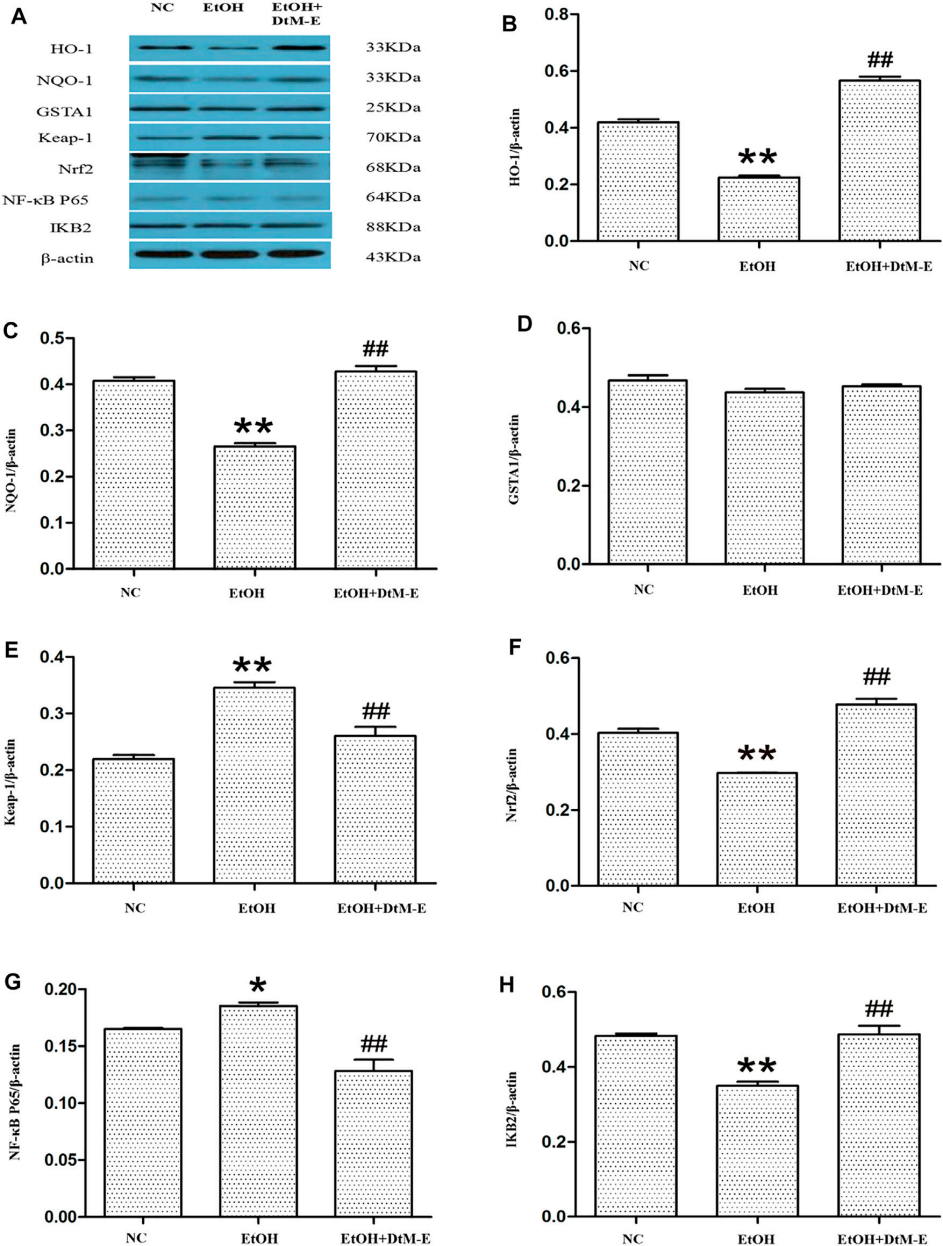
The Western blot of Nrf2 and NF-κB signaling **(A)**. Quantification of HO-1 **(B)**, NQO-1 **(C)**, GSTA1 **(D)**, Keap-1 **(E)**, Nrf2 **(F)**, NF-κB P65 **(G)**, and IKB2 **(H)** expression. All data are presented as mean ± SD (*n* = 6). **p < 0.01, *p < 0.05 (*vs*. control group); ^##^ *p *< 0.01 (*vs*. model group).

**FIGURE 6:**
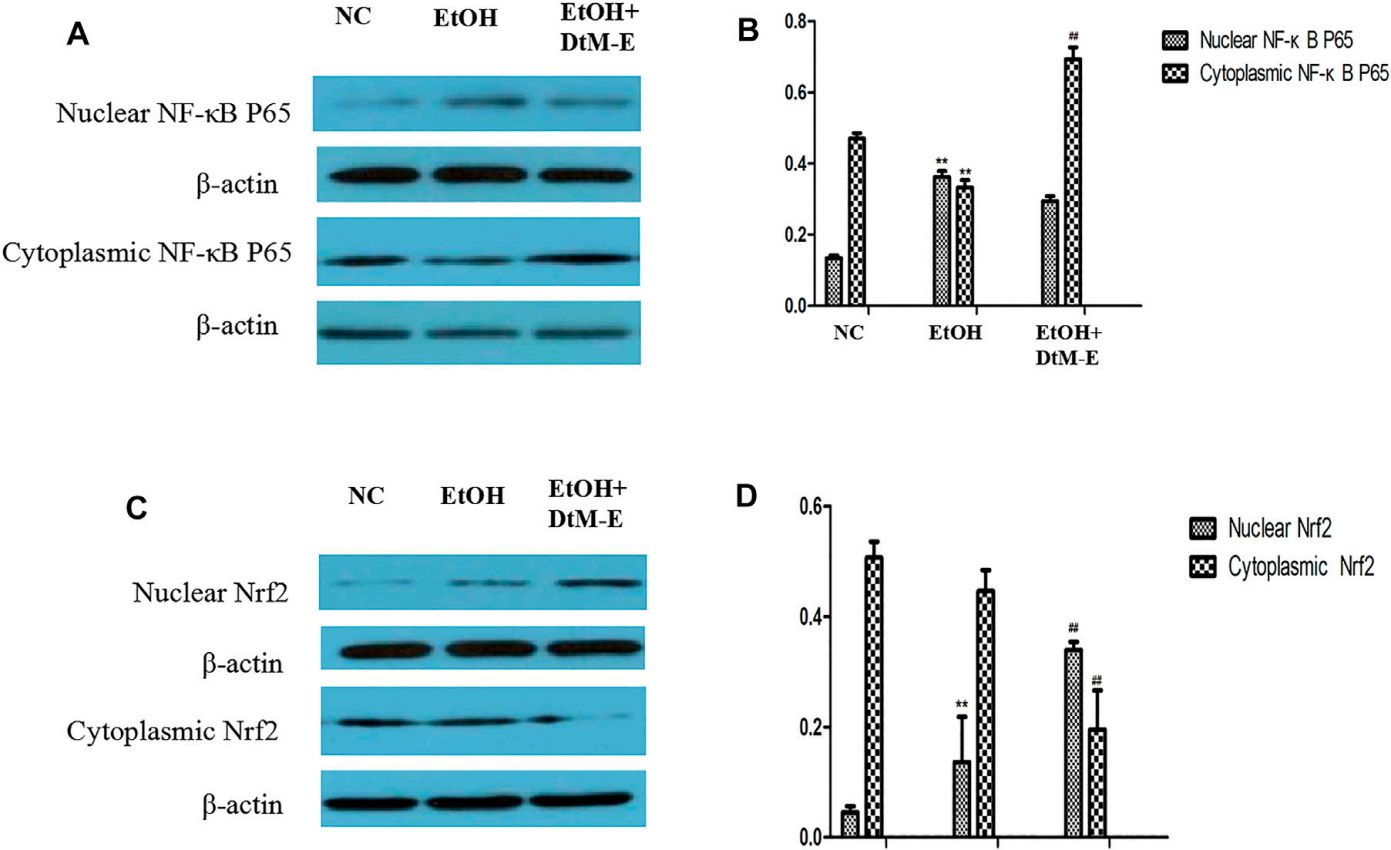
The Western blot of nuclear and cytoplasmic NF-κBP65 **(A)**, nuclear and cytoplasmic Nrf2 **(C)**. Quantification of nuclear and cytoplasmic NF-κBP65 **(B)**, nuclear and cytoplasmic Nrf2 **(D)** expression. All data are presented as mean ± SD (*n* = 6). **p < 0.01, (*vs*. control group); ^##^ *p *< 0.01 (*vs*. model group).

The results (Figure 5) showed that compared with the NC group, alcohol downregulated the expression of Nrf2, HO-1, NQO-1, and IKB2, while it upregulated the expression of Keap-1 and NF-κB P65. Compared with the model group, *DtM-E* upregulated the expression of Nrf2, HO-1, NQO-1, and IKB2, while it downregulated the expression of Keap-1and NF-κB P65. As shown in Figure 6, the expression of cytoplasmic NF-κB P65 was decreased in the model group and increased in the DtM-E group, while the expression of nuclear NF-κB P65 was increased in the model group and decreased in the DtM-E group. The expression of cytoplasmic Nrf2 was slightly decreased in the model group and decreased in the DtM-E group, while the expression of nuclear Nrf2 was increased in the model group and the DtM-E group.

## Discussion

Alcoholic liver disease (ALD) refers to the damages to the liver and its functions due to alcohol overconsumption. Excessive and continued alcohol use results in fatty changes in the liver, which can develop into hepatitis, fibrosis, cirrhosis, and even liver failure and hepatocellular carcinoma (Farooq and Bataller, 2016). Alcohol-induced liver cancer ranks first among all causes of liver cancer mortality (Singal and Mathurin, 2021). Given the high morbidity, mortality, and the progressive nature of ALD, finding effective interventions is essential.

In this study, we observed the amelioration of three extracts of *DTM* against ALD *in vitro* and *in vivo*, and the engaged mechanism with the aim of providing a potential treatment for ALD. When the liver is damaged by alcohol intake, the cells rupture, and the levels of ALT and AST in the cells enter the serum, resulting in an increase in the levels of both. At the same time, when alcohol is metabolized by enzymes, it can induce the production of ROS in cells and consume a lot of reducing substances, such as GSH, which leads to the production of abnormal free radicals in cells and gradually accumulate and diffuse, causing oxidative stress, and then causing cell-related structure destruction and the function to be abnormal (Kawaratani et al., 2017; Waris et al., 2020). Therefore, the content of GSH, SOD, and CAT is an important factor to measure the ability of antioxidation, and the content of MDA can reflect the degree of oxidative damage and cell injury (Khoubnasabjafari et al., 2015). Because of the imbalance between lipid degradation and synthesis, hepatic steatosis is a risk factor for liver pathological development. TG, TC, HDL-C, and LDL-C are often used as biomarkers of hepatic steatosis. Therefore, the above indexes were listed as the key liver function indexes in the alcohol induction experiment.

To find the ideal experimental models *in vivo* that recapitulate the full spectrum of ALD is critical in our ability to identify new mechanisms and therapeutic targets. However, over the last decades, a great variety of animal models for ALD have been developed with different limitations (Lamas-Paz et al., 2018; Dastidar et al., 2018; Seitz et al., 2018). In this study, 40% ethanol administered by oral gavage was used to induce an acute ALD mice model. The results in the model group showed that the serum contents of AST, ALT, TG, TC, LDL-C, and MDA in liver tissue were significantly increased, while the serum contents of HDL-C and GSH-Px, SOD, CAT, and ADH in liver tissue were significantly decreased. The hepatic lobules were found to be structurally disordered, the hepatocytes were swollen and showed balloon-like degeneration, and a few hepatocyte nuclei were pyknosis. The intralobular lymphocyte infiltration, fat deposits, and blue collagen fibers also appeared. The above biochemical and pathological indexes revealed that the acute ALD mice model was successfully established. Three extracts of *DTM* ameliorated alcohol-induced liver injury in different degrees, and *DtM-E* worked best. After *DtM-E* treatment, compared with the model group, serum liver function enzyme, serum lipid, lipid peroxidation indexes, and alcohol-metabolizing enzyme in liver tissues tended to be normal. In addition, the liver tissues of ALD mice also showed significant improvement in pathology.

In this study, ethanol was selected to induce an alcoholic liver injury cell model, and the effects of different extracts of *DtM* on normal and EtOH*-*induced cells were also studied. The results showed that when the concentration of ethanol was 200 μM, the cell activity was significantly decreased (70.77 ± 5.53), and the cell model of alcoholic liver injury was successfully established. Three extracts of *DTM* had no effect on normal cell vitality, while EtOH*-*induced cell vitality improved in varying degrees; *DtM-E* worked best at a concentration of 40 μg/ml, which showed that *DtM-E* had a protective effect on ethanol-induced hepatocyte injury and was dose related.

The pathogenesis of ALD is extremely complicated and involves immune cells, adipose tissues, and genetic diversity (M Omar Farooq and Bataller, 2016). Transcription factors are regulatory factors that affect the expression of target genes by binding to DNA sequences of gene promoters during cell transcription with a number of more than 1,600 (Lambert et al., 2018). It has been shown that some transcription factors play an important role in regulating various aspects and development of ALD in various ways. Based on the literature, six ALD-related transcription factors (He and Karin, 2011; Nishiyama et al., 2012; Davis et al., 2017; Sun et al., 2018; Schulien et al., 2019; Nowak and Relja, 2020) (NRF2, NF-κB, STAT3, AP-1, CREB, and HIF-1α) were selected in this study. They played complex roles in hepatic inflammation, fibrosis, and hepatocarcinoma *via* the induction of its target genes. The PCR results showed that, compared with the model group, *DtM-E* upregulated the expression of Nrf2 and downregulated the expression of NF-κB dramatically. To further explore the mechanism of *DtM-E* in alleviating ALD *via* Nrf2 and NF-κB, the expression levels of Nrf2 and NF-κB negative regulatory proteins (Keap1, IKB2) and the key downstream enzyme genes (HO-1, NQO1, GSTA1) were detected by qRT-PCR method. In Figure 4B, after *DtM-E* treatment, Nrf2 was activated, and the expression of HO-1, NQO-1, and GSTA1 was also upregulated, while NF-κB was inhibited, and the expression of Keap-1 and NF-κB P65 was downregulated when compared with the model group. The Western blot results were consistent with that of PCR.

Nuclear translocation is one of the main transduction pathways involved in many intracellular signaling pathways and regulation of genetic information (Liu et al., 2016). In this study, the regulation effects of *DtM-E* on NF-κB p65 and Nrf2 nuclear translocation were discussed, and treatment with *DtM-E* induced a substantial localization of Nrf2 to the nucleus and NF-κB p65 to the cytoplasm in cells.

The results suggest that *DtM-E* has a protective effect on ALD, and this may be related to the inhibition of oxidative stress–inflammatory injury. Oxidant stress plays a dominant role in the clinical and pathological spectrum of ALD. Many studies have shown that the oxidative metabolites of ethanol, such as ROS, are key to the progression of hepatic inflammation, fibrosis, and hepatocarcinogenesis (Ceni et al., 2014; Li et al., 2015). In this study, after *DtM-E* treatment, Nrf2 is activated, located to the nucleus, and bound to the ARE element of the downstream target gene, inducing a large number of cell protective proteins, such as GST, NQO1, HO-1, and GSH to prevent ROS-induced tissue damage. Meanwhile, *DtM-E* inhibits the expression of Keap1, which is the negative regulatory protein of Nrf2. ALD starts with steatosis, followed by inflammatory changes induced by ROS generated by the metabolism of alcohol. *DtM-E* also inhibits the nuclear translocation and expression of NF-κB P65, reducing the inflammatory changes induced by proinflammatory cytokines. There is crosstalk between the NF-κB and Nrf2 signaling pathways; the activation of Nrf2 can prevent inflammation (Ramos Tovar and Muriel, 2020). Therefore, the protective effect of *DtM-E* on ALD is the result of the joint regulation of NF-κB and Nrf2 signaling pathways.

## Data Availability

The original contributions presented in the study are included in the article/Supplementary Material, further inquiries can be directed to the corresponding authors.
